# Complete genome sequence of marine *Enterobacter cloacae* BARC_01

**DOI:** 10.1128/mra.01417-25

**Published:** 2026-02-18

**Authors:** Atif Khan, Hiren M. Joshi

**Affiliations:** 1 Water & Steam Chemistry Division, BARC Facilities, Kalpakkam, Tamil Nadu, India; 2 Homi Bhabha National Institute 232022, Mumbai, Maharashtra, India; Montana State University, Bozeman, Montana, USA

**Keywords:** *Entrobacter cloacae*, biocide resistance, antibiotic resistance, antropogenic contamination

## Abstract

We report the complete genome (CP183059.1) and plasmid (CP183058.1) sequences of marine *Enterobacter cloacae* BARC_01, isolated from a chlorinated marine cooling water system. The strain has more than 98% average amino acid and average nucleotide identity to the terrestrial pathogenic reference strain *E. cloacae* ATCC 13047.

## ANNOUNCEMENT

*Enterobacter cloacae* is a clinically significant member of the ESKAPE group, frequently implicated in hospital-acquired infections such as pneumonia and sepsis (1, 2). While clinical *E. cloacae* complex (ECC) members are well documented, environmental isolates remain understudied. We report the complete chromosome and plasmid sequences of *E. cloacae* BARC_01 (rMLST matching), isolated from coastal seawater near an operational power plant in India (12°31′25.7232″ N, 80°9′24.5340″ E). This strain harbors an extensive repertoire of resistance determinants, closely resembling the pathogenic reference strain *E. cloacae* ATCC 13047.

The single colony of isolated bacteria was inoculated in 100 mL Zobell Marine broth (Hi-Media) and incubated at 30°C and 100 rpm in an incubator shaker for 24 hours. DNA was extracted using the DNeasy PowerLyzer Microbial Kit (Qiagen). Purity and concentration were assessed via the Tecan nanoplate reader and Qubit High Sensitivity dsDNA Assay (Thermo), respectively. One microgram of isolated DNA was directly used for library preparation using the Native Barcoding Kit 24 (v14) (SQK-NBD114.24, Oxford Nanopore Technologies) and sequenced on a MinION flow cell (R9.4.1). Base calling was performed using Guppy (v6.5.7) in high-accuracy mode.

**Fig 1:**
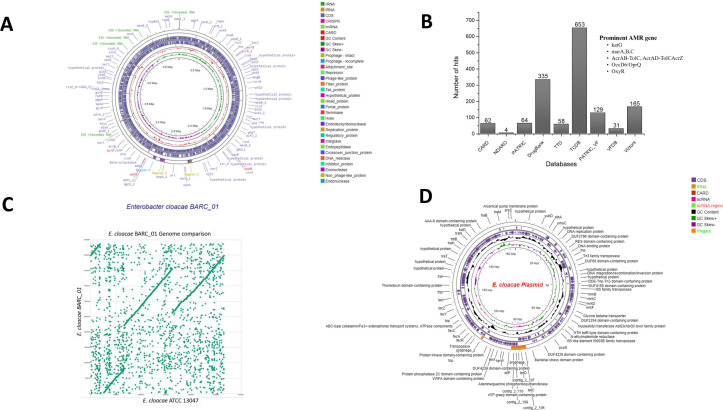
(**A**) Circular genome of *Enterobacter cloacae* BARC_01. (**B**) Number of positive hits against various antimicrobial resistance databases and major genes found in the genome of *E. cloacae* BARC_01. (**C**) Genome comparison (dot plot) of *E. cloacae* BARC_01 and *E. cloacae* ATCC 13047 (reference strain). (**D**) Circular plasmid of *E. cloacae* BARC_01.

We obtained 22,589 reads (mean length 4.9 kb, N50 12.9 kb). The adapter was trimmed using the Porechop utility and *de novo* assembled using Flye (v2.9.6) (3) and polished via the Medaka pipeline (v2.1.1) (https://github.com/nanoporetech/medaka). Assembly quality was verified by BUSCO (C: 95.2%, F: 3.2%, M: 1.6%; *n* = 124). Annotation was performed using Prokka (v1.14.6) and the RAST server (4, 5). Circular genome visualization (Fig. 1A) was generated using Proksee (6). Antimicrobial resistance genes, virulence factors, and transporters were identified using the BV-BRC platform (6) against CARD, PATRIC, VFDB, and TCDB databases (Fig. 1B).

The plasmid was found to be 163,703 bp in length with a 53% GC content and contained 116 putative coding sequences, including conjugation clusters and heavy metal resistance genes (Fig. 1D). Its size, GC content, and repertoire of mobile genetic elements and resistance genes were characteristic of IncHI-like megaplasmids previously described in clinical ECC isolates (7).

Comparative genomics against *E. cloacae* ATCC 13047 (CP001918.1) (Table 1) revealed 98% average nucleotide identity and 99.3% average amino acid. MUMmer (v4.0.0) dot-plot alignment (Fig. 1C) confirmed high structural synteny with minor strain-specific rearrangements (8). These findings suggest *E.cloacae* BARC_01 might be of terrestrial origine and entred in to the marine environment via anthropogenic activity, retaining significant antimicrobial resistance potential.

**TABLE 1:** 

Feature category	*E. cloacae* BARC_01	Reference genome (*E. cloacae* ATCC 13047/RefSeq NC_015663)
Genome size/CDS count	5,359 CDSs; 5,623 total features	~5,200 to 5,500 CDSs
RNA elements	111 RNA genes	100–120 RNA
Hypothetical proteins	~11.3% of CDS	10%–18% typical
CRISPR–Cas content	*7 cas* genes, 38 spacers, 40 repeats	Presence variable; many reference strains have truncated CRISPR systems
β-Lactamase genes (AmpC family)	5 predicted loci	1–5, depending on plasmid load
Efflux pump genes	~100 genes	90–120 genes are typical
Mobile elements/IS elements	50 transposase-like genes	Dozens common
Regulatory protein families (LysR/AraC/TetR)	LysR: 55 • AraC: 25 • TetR: 6	Broadly enriched in Enterobacteriaceae

## Data Availability

All raw sequence data have been deposited in the National Center for Biotechnology Information under BioProject PRJNA1216755 and BioSample SAMN46445679, with corresponding Sequence Read Archive accession number SRR36283896. All the sequence data have been submitted to GenBank, with accession numbers CP183059.1 (genome) and CP183058.1 (plasmid).
